# Supplementary material for: Marketplace shrimp mislabeling in North Carolina
Source: PLoS One. 2020 Mar 12;15(3):e0229512. doi: 10.1371/journal.pone.0229512 (PMC7067418; doi:10.1371/journal.pone.0229512)
Supplement: Attachment — Submitted filename: PLoS editorial comment_Mislabeling shrimp.docx [file pone.0229512.s001.docx]

PLoS editorial comment_Mislabeling shrimp in NC

Overall, a nicely written manuscript with interesting results. The references are not formatted correctly in the text and should be reference numbers. I thought the Discussion could say a bit more like what happens next, are there any legal ramifications for the vendors selling mislabelled shrimp, what consumers can do etc. Just a bit more would be useful.

Line 26: Replace the colon with a comma

Line 28: Should be ‘mangrove forests’

Line 29: This is confusing as written, how is seafood importation a negative consequence of shrimp farming?

Lines 29 and 30: Replace the dashes with commas

Line 35: The authors use both United States and U.S. in the manuscript and should be consistent. I would write United States (U.S.) here and use U.S. throughout the rest of the paper.

Line 43: The term ‘fishers’ is preferable to ‘fishermen’; write as ‘82.9% higher than’

Line 45: Write as ‘valued at USD $29.6 million’ here so it is clear what currency is being reported.

Line 49: ‘fishers’

Line 56” Should be ‘outreach programs’

Line 65: Write as ‘species in high demand’

Line 75: This is awkward as written ‘enforce mislabelling practices;…..that sounds like supporting mislabelling practices so please rephrase.

Line 97: Write as ‘(31 inland vendors and 29 coastal vendors)’

Line 100: Replace ‘yet’ with ‘but’

Line 105: Insert ‘a’ before ‘failure’

Line 125: What does ‘ETON’ stand for?

Line 126: Write as ‘known samples on the’

Line 136” The full scientific name white shrimp has already been provided so abbreviate here

Line 137: These were defined as white shrimp on line 85 so a bit repetitive

Line 140: Should be ‘commonly farmed’

Line 156: In review references should not be in the reference list. See the submission guidelines for more information.

Line 160: Don’t need to define the abbreviation ‘NOAA’ here as it is not used again in the paper except for in the references, which will change to reference number in the text.

Line 172: Spelling error ‘shrimp’

Line 186: Need a comma after ‘Surprisingly’

Line 189: Full stop after ‘mislabeling’ and start the next sentence ‘Revisiting vendors’

Line 190: Should be ‘changes’

Line 191: Delete ‘yet’ as it makes no sense here

Line 193: Replace ‘holistic’ with ‘complete’
