# Supplementary material for: Marketplace shrimp mislabeling in North Carolina
Source: PLoS One. 2020 Mar 12;15(3):e0229512. doi: 10.1371/journal.pone.0229512 (PMC7067418; doi:10.1371/journal.pone.0229512)
Supplement: Attachment — Submitted filename: PLOS ONE Shrimp response letter.doc [file pone.0229512.s002.doc]

Department of Biology

Coker Hall

Campus Box 3280

Chapel Hill, NC 27599-3280 USA

Email: [jbruno@unc.edu](mailto:jbruno@unc.edu)

Twitter: @johnfbruno

Web: [www.johnfbruno.com](http://www.johnfbruno.com/)

College of Arts & Sciences

February 6, 2020

Dear Dr. Patterson,

Thank you for considering our manuscript. We greatly appreciate the time and effort you and the reviewers have put into improving our work. And we apologize for the delay in resubmitting.

The suggested edits were very helpful. We have completely revised the manuscript, and below we respond (in bold) to all the feedback you have provided and have explained the changes we have made.

Sincerely,

John Bruno (on behalf of all the authors)

John F Bruno, Ph.D.

Professor

PONE-D-19-24180

Marketplace Shrimp Mislabeling in North Carolina

PLOS ONE

Dear Dr. Bruno,

Thank you for submitting your manuscript to PLOS ONE. After careful consideration, we feel that it has merit but does not fully meet PLOS ONE’s publication criteria as it currently stands. Therefore, we invite you to submit a revised version of the manuscript that addresses the points raised during the review process.

I found this to be a well written and interesting manuscript and both reviewers agreed. Overall, the text was clear and concise and the results timely. Both reviewers have provided relatively minor comments to improve the clarity of the paper and I agree. I have some generally minor editorial and formatting comments that need to be addressed. I do think that the methods need to be spelled out a bit more and the discussion could do a bit more to clarify the ramifications of the results. But I think these changes can be made pretty easily and both reviewers have provided detailed comments to assist the authors in their revision. I strongly encourage the authors to consider and address all the comments provided.

We would appreciate receiving your revised manuscript by Nov 10 2019 11:59PM. When you are ready to submit your revision, log on to <https://www.editorialmanager.com/pone/> and select the 'Submissions Needing Revision' folder to locate your manuscript file.

If you would like to make changes to your financial disclosure, please include your updated statement in your cover letter.

To enhance the reproducibility of your results, we recommend that if applicable you deposit your laboratory protocols in [protocols.io](http://protocols.io/), where a protocol can be assigned its own identifier (DOI) such that it can be cited independently in the future. For instructions see: [http://journals.plos.org/plosone/s/submission-guidelines#loc-laboratory-protocols](http://journals.plos.org/plosone/s/submission-guidelines#loc-laboratory-protocols)

Please include the following items when submitting your revised manuscript:

• A rebuttal letter that responds to each point raised by the academic editor and reviewer(s). This letter should be uploaded as separate file and labeled 'Response to Reviewers'.

• A marked-up copy of your manuscript that highlights changes made to the original version. This file should be uploaded as separate file and labeled 'Revised Manuscript with Track Changes'.

• An unmarked version of your revised paper without tracked changes. This file should be uploaded as separate file and labeled 'Manuscript'.

Please note while forming your response, if your article is accepted, you may have the opportunity to make the peer review history publicly available. The record will include editor decision letters (with reviews) and your responses to reviewer comments. If eligible, we will contact you to opt in or out.

We look forward to receiving your revised manuscript.

Kind regards,

Heather M. Patterson, Ph.D.

Academic Editor

PLOS ONE

Journal Requirements:

When submitting your revision, we need you to address these additional requirements.

1. Please ensure that your manuscript meets PLOS ONE's style requirements, including those for file naming. The PLOS ONE style templates can be found at

<http://www.journals.plos.org/plosone/s/file?id=wjVg/PLOSOne_formatting_sample_main_body.pdf>and <http://www.journals.plos.org/plosone/s/file?id=ba62/PLOSOne_formatting_sample_title_authors_affiliations.pdf>

2. In your Methods section, please provide additional details regarding the shrimps used in your study and ensure you have described the source. For more information regarding PLOS' policy on materials sharing and reporting, see [https://journals.plos.org/plosone/s/materials-and-software-sharing#loc-sharing-materials](https://journals.plos.org/plosone/s/materials-and-software-sharing#loc-sharing-materials).

3. Have the authors made all data underlying the findings in their manuscript fully available?

**We are sharing all of our sequence data in a spreadsheet that includes sample number, collection location/date, BLAST results, and DNA results. Additionally, we are in the process of uploading our sequences to GenBank. Our intention has been to make everything available and be totally transparent, however, we have experienced some challenges with GenBank (but are working through them).**

Reviewer #1: This is a very well-written and interesting paper for which I have little to say or add. I think it makes a great contribution to the literature on mislabeling, in fact, I haven’t seen an empirical paper as useful and well-framed in this field for some time.

**Thank you! The undergrad students that did the work and wrote the paper were thrilled with this comment!**

The most important comment I have is that reporting only the top Blast hit isn’t convincing enough (see below). What follows are some specific comments organized by line number.

Lines 128-130: The Blast results can be more convincing if something like the top X hits were all the same species and the next best hit that was not that species was <96% or something like that. There are many mistakes in GenBank, so suppose a top hit was misidentified by whoever submitted the sequence. Putting the sequences in a phylogeny can be helpful to, to see that the unknowns sit in monophyletic clades that are all the same species. BOLD might be worth checking because one can have much more confidence in the ID there.

**Thank you. We added “Additionally, we recorded the percent identity of the next species down in the BLAST results.” to this paragraph and included this data with our sample BLAST results.**

Lines 38-39: Why “Despite” - seems vague, perhaps implying that the domestic market could supply domestic demand. I doubt that, but the manuscript should more clearly establish that if it’s true or tweak this sentence.

**We are not implying that; we were simply noting that Americans eat a lot of shrimp, some of it is harvested in the US, but most of it is imported, ie, just basic background info. We revised this sentence to: *Shrimp is the most popular seafood in the United States. Shrimp represents a quarter of America’s annual per capita seafood consumption and the average American eats about five pounds of shrimp each year (NMFS, 2018). This results in over one billion pounds of shrimp consumed annually in the United States alone, 80% of which is imported (NMFS, 2018). In 2018, the U.S. imported 136 million pounds of shrimp, primarily from Indonesia, India, and Ecuador, which accounted for 33% of all reported seafood imports (NMFS, 2019; NMFS, 2018).***

Lines 48-52: This seems likely, but I suppose this suggests a causal relationship that isn’t necessarily there. What other factors might control declines in landings, licenses, and processors? Regulations?

**We revised this sentence to: *“However, pressure from regulations and imported seafood products are believed to be at least partially responsible for recent declines in North Carolina’s seafood industry (Kros and Rowe, 2013).”***

Lines 70-71: Oceana has a more recent study: Warner, K., Roberts, W., Mustain, P., Lowell, B., & Swain, M. (2019). Casting a Wider Net: More Action Needed to Stop Seafood Fraud in the United States, (March), 19. <https://usa.oceana.org/publications/reports/casting-wider-net-more-action-needed-stop-seafood-fraud-united-states>

**Thank you - citation added.**

Line 93: How was certification by NC Catch determined by the authors? What I’m wondering is if the claim itself might often be fraudulent.

**Our view is that the claim is vague, so technically not fraudulent. They don’t directly claim to certify catch - all they do is report vendors that claim (without verification) to sell *some* locally caught product. It’s obviously misleading to the consumer, who could reasonably conclude that NC Catch is some type of state certification program. We are working with them to revise their policies and objectives! They were caught off guard by the results.**

Line 111: Qiagen misspelled.

**Corrected.**

Line 119: I looked at Willette and Ivanova and I don't understand the use of 4 primers (those papers do not include them). Were different pairs of primers used on different specimens or was this some sort of nested PCR?

**Thank you. We corrected the citation to Ivanova et al. (2007), which includes the primers that we used for this study.**

Line 139: I would be as explicit as possible about what is meant by “native.” “Does not occur in the Western Atlantic” or “has never been reported from the Western Atlantic…”

**Thank you. We revised this sentence as: *“does not occur in the western Atlantic.”***

Line 146: Is each sample truly independent? If two were bought on the same day from the same vendor (pulled out of the same bin), probably not. It sounds like multiple samples from the same bin only resulted from revisiting the same vendor, but the Methods need to be more explicit about that.

**Agreed. Although we sequenced 106 shrimp, we sampled only 60 vendors, and we think that is the true sample size. Of those 60, 11 were sampled more than once (i.e, in more than one season or year) and from 9 (of the 60) we collected and sequenced more than one “variety” of shrimp (e.g., of different sizes, deveined or not, etc.). (Note we also always collected 3 or more shrimp of every variety from every vendor. For some of the vendors, we processed multiple shrimp to test whether there was a mixture of mis- and correctly labeled shrimp of a given variety, mixed together in a single container - usually a metal pan. We never found any variance among replicate shrimp, i.e., sample identity was always concordant, so we didn’t continue to process every replicate sample for most the samples collected in the later months and in 2018. None of this replication is included in the paper and analysis. This was done as a preliminary analysis to assess how much replication was needed per vendor visit.)**

**We have expanded the explanation of the sampling scheme in the Methods section:**

***“We purchased shrimp from 60 vendors across North Carolina (31 were inland vendors and 29 were coastal) in spring (May 18 through June 5) and fall (September 20 through November 4) of 2017 and during the spring of 2018 (Fig. 1). We defined inland vendors as ones located in a land-locked county. The cost ($/lb) for each sample collected was recorded.***

***Twenty-three of the 60 vendors were sampled in multiple years and / or seasons, i.e., to quantify labeling consistency. For 10 vendors, we sampled more than one “variety” of shrimp on a given sampling date. Such varieties (e.g., of different sizes, deveined or not, etc.) were typically displayed in separate containers, usually a metal pan. For every sampling visit, we obtained three separate shrimp from each sampled variety and froze these in 2-mL plastic screw cap tubes.***

***For most of the vendors sampled in the spring of 2017, we processed multiple shrimp to test whether there was a mixture of mis- and correctly-labeled products of a given variety, mixed together in a single container. We never found any variance among replicate shrimp (i.e., sample identity was always concordant), so we did not continue to process every replicate for samples collected in the fall of 2017 and in 2018. The exception was when we are not able to successfully sequence the first sample.”***

I see no statements about the sequences having been deposited in GenBank.

***We are in the process of uploading our sequences to GenBank. Additionally, we are sharing all of our sequence data in a spreadsheet that includes sample number, collection location/date, BLAST results, and DNA results.***

Reviewer #2: The study by Korzik et al. describes the first investigation of shrimp mislabeling or species substitution in North Carolina. The study was apparently conducted over several semesters, spring and fall, as part of undergraduate training in DNA forensics. The authors point out that shrimp are an important and valuable North Carolina fishery and that mislabeling may result in mistrust of the local product. The authors collected and successfully analyzed to the species level via DNA barcoding 106 shrimp products from 60 retail vendors located in coastal and inland counties that were certified sold as “certified” “local” shrimp. Ten of the vendors were sampled more than one time. Results of the DNA species identity testing revealed that roughly one third of the products and vendors sold imported farmed shrimp as local wild-caught shrimp, remarkedly consistent with results from a previous nationwide study on shrimp mislabeling from 2014. Furthermore, there was no statistically significant difference between the mislabeling rates between inland and coastal counties. The results of repeat testing of vendors were unclear as presented. The study also showed that mislabeled products were sold at a cheaper price compared to honestly labeled products, but this result was not discussed.

**We added text describing this result to the Discussion**

The study lacks a description of statistical methods used and a Conclusion section.

**Statistical methods were added, however, we don’t believe a short report like this requires a distinct Conclusions section in addition to the wrap up paragraph we already have at the end of the Discussion.**

It could also use another table or graph to explain the repeated sampling of some vendors.

**We agree this was unclear. We revised the Methods section to more clearly explain the sampling design (see above).**

The authors should note and cite other shrimp mislabeling studies to put their findings in context and expand on what the NC Catch certification entails. However, with these and other revisions noted below, this study should expand our sparse knowledge of shrimp mislabeling and its geographic reach, particularly in a state not previously investigated, yet known for its shrimp fishery. The study also nicely outlines the cultural and economic value of the shrimp fishery in NC and the current struggles the domestic shrimp industry faces.

**See responses below.**

Specific comments:

Abstract:

Line 27: As written, implies that that all the substituted whiteleg shrimp were from the eastern Pacific, when in fact they are farmed all over the world. Please clarify that the aquacultured species is native to that region.

**Thank you. We altered this sentence to say: *“Roughly one third of product incorrectly marketed as “local” was in fact whiteleg shrimp: an imported and globally farmed species native to the eastern Pacific, not found in North Carolina waters.”***

Line 28: What is the negative ecosystem consequence of seafood importation? Please clarify or reword.

**Agreed. Shrimp farming, and not importation, is what has a negative ecosystem impact. We cut “and seafood importation” from the relevant section in the Abstract.**

Introduction:

58: Please explain in more detail what the NC Catch certification entails. Does the advocacy organization spot check supply records or have other means of verifying that vendors are selling local products? This might be a topic for the Conclusion section.

**Done. Also see above.**

63: “Mislabeling” of seafood could encompass misstating the country of origin or correct weight. Please clarify that you are defining it as species substitution.

**We agree, however, in this study we defined mislabeled as: *“For this study we defined seafood mislabeling as occurring when a species is substituted with another type of seafood, including ones of lower economic value (Marko et al., 2004).”***

70-71: The Warner et al. 2014 citation concerns shrimp mislabeling only, not “seafood” in general, while the Warner et al. 2016 is correctly cited as referring to all seafood. Please clarify.

**Thank you. We altered this sentence to say: *“According to a 2019 study, 21% of seafood is mislabeled in the United States, and 30% is mislabeled worldwide (Oceana, 2019; Warner et al., 2016).”***

72-74. Please expand with examples or explain how mislabeling leads to the negative consequences described, such as human rights abuses and overfishing.

**We expand on the negative consequences of mislabeling, including human rights abuses and overfishing, in our Discussion section.**

74-75: I believe that only testing or traceability along the entire seafood supply chain can pinpoint the source of mislabeling. Consider revising this sentence to clarify.

**We agree and cut this sentence.**

80-81: The authors should cite the other shrimp studies conducted.

**Remarkably, only a few studies, globally, have tested the mislabeling of shrimp. A recently published global meta-analysis of 141 studies of seafood mislabeling (Luque and Donlan 2019) reported:** “**Our analysis revealed important seafood products where data is severely lacking with respect to mislabeling. For example, only nine studies have sampled the shrimp family (Penaeidae). Yet, global shrimp production in 2016 was 8.6 million tonnes (FAO, 2018). This includes a single sample from a single study from the United States, where shrimp is one the most consumed seafood products (Khaksar et al., 2015a; NMFS, 2016). An unpublished report also sampled shrimp (n = 143 samples) in the United States (Warner et al., 2014). While mislabeling was documented, we were unable to extract the information needed to include it in our meta-analysis.”**

**Luque GM and Donlan CJ. The characterization of seafood mislabeling: A global meta-analysis.**

**Biological Conservation. 2019; 236: 556-570. doi.org/10.1016/j.biocon.2019.04.006**

**Warner, K., Golden, R., Lowell, B., Disla, C., Savitz, J., Hirshfield, M., 2014. Shrimp: Oceana Reveals Misrepresentation of America's Favorite Seafood. Oceana, Washington DC**

**Thus, unbelievably, this would be the first published assessment of shrimp mislabeling in the US.**

Materials and Methods:

97-98: Describe whether shrimp were collected during the shrimp fishery season in NC, since the authors speculate later that this may be a factor in mislabeling (e.g. line 192).

**See below.**

133: Add a small section on statistical methods used

**Done.**

Results:

140: Reword “It is common farmed”. L. vannamei is the most commonly farmed shrimp species worldwide.

**Thank you. We altered the sentence to say: *“It is the most commonly farmed species worldwide, especially in coastal Ecuador (Fofonoff et al., 2018).”***

143-144: The sentence starting with “Of the ten..” is unclear. Consider representing the repeat testing of vendors in a table or graph to help the reader understand the results of this testing.

**Thank you. We revised this portion of our Methods section to be more clear (see above).**

Discussion:

It would help to discuss the price difference observed between correctly labeled and mislabeled shrimp. Was the lower cost of mislabeled shrimp close to, higher or lower than the average price of farmed shrimp, sold under the correct label of farmed shrimp in NC?

**Done, added.**

171-173: Sentence starting with “Shrimp farms…” needs a reference.

**Agreed: we added (Avila, 2012).**

177: Consider adding FDA import refusal data for shrimp as an updated reference for this section and discuss.

**The FDA Import Refusal Database lists monthly refused imports by class (i.e. seafood products) and company rather than by product. Because the number of imported shrimp refused differs each month and by company, we feel that including this data could stray away from our core research question, as our study focuses on shrimp that have made it to the North Carolina marketplace and are labeled as "local."**

191-192: Begs the question if any sampling occurred when the shrimp fishery was closed or out of season. Please clarify here and in the Methods section.

**Shrimping in North Carolina is not bound by specific dates and is instead determined by the Division of Marine Fisheries Director and is based on average shrimp size. In the ocean, shrimping is essentially always open, as predominantly large shrimp are caught there. During the winter, shrimping declines because the shrimp migrate from that area during those months. In the North Carolina estuaries and sounds, shrimping season is regionally specific, but the season opens when the shrimp hit ~30-35 count (heads on). However, even that size threshold can vary at the Division of Marine Fisheries Director’s discretion. In general, shrimping opens in early spring and goes until the shrimp migrate out of the estuaries in winter, so the North Carolina shrimping season would be open during all of our sampling times.**

195: Consider adding a Conclusion section on how to improve the NC catch certification and discuss whether a lower price for local shrimp may be “too good to be true” and an indication of potential mislabeling. For example, after the prevalent grouper mislabeling incidents in Florida, the state responded in marketing that conveyed what an honestly labeled grouper dish should cost. See: <https://www.fdacs.gov/Food-Nutrition/Food-Safety-Resources/Mislabeling-Seafood-Products-Is-Illegal>

**Great ideas.**
